# Nascent flagellar basal bodies are immobilized by rod assembly in *Bacillus subtilis*

**DOI:** 10.1128/mbio.00530-25

**Published:** 2025-05-21

**Authors:** Caroline M. Dunn, Daniel J. Foust, Yongqiang Gao, Julie S. Biteen, Sidney L. Shaw, Daniel B. Kearns

**Affiliations:** 1 Department of Biology, Indiana University 123993, Bloomington, Indiana, USA; 2 Department of Chemistry, University of Michigan 205700, Ann Arbor, Michigan, USA; 3 Department of Microbiology, Harvard Medical School 1811 , Boston, Massachusetts, USA; Fred Hutchinson Cancer Center, Seattle, Washington, USA

**Keywords:** flagella, FliM, membrane, patterning, motility, FRAP, microscopy, TIRF, particle-tracking

## Abstract

Flagella are complex, trans-envelope nanomachines that localize in species-specific patterns on the cell surface. Here, we study the localization dynamics of the earliest stage of basal body formation in *Bacillus subtilis* using a fluorescent fusion to the C-ring protein FliM. We find that *B. subtilis* basal bodies do not exhibit dynamic subunit exchange and are largely stationary at steady state, consistent with flagellar assembly through the peptidoglycan (PG). However, rare mobile basal bodies were observed, and the prevalence of mobile basal bodies is elevated both early in basal body assembly and when the rod is mutated. Thus, basal body mobility is a precursor to patterning, and we propose that rod polymerization probes the PG superstructure for pores of sufficient diameter to permit rod transit. Furthermore, mutation of the rod disrupts basal body patterning in a way that phenocopies mutation of the cytoplasmic flagellar patterning protein FlhF. We infer that rod synthesis and the cytoplasmic regulators coordinate flagellar assembly by interpreting a grid-like pattern of pores, pre-existent in the PG.

**IMPORTANCE:**

Bacteria insert flagella in a species-specific pattern on the cell body, but how patterns are achieved is poorly understood. In bacteria with a single polar flagellum, a marker protein localizes to the cell pole and nucleates the assembly of the flagellum at that site. *Bacillus subtilis* assembles ~25 basal bodies over the length of the cell in a grid-like pattern and lacks proteins required for their polar targeting. Here, we show that *B. subtilis* basal bodies are mobile soon after assembly and become immobilized when the flagellar rod transits the peptidoglycan (PG) wall. Moreover, defects in the flagellar rod lead to a more-random distribution of flagella and an increase in polar basal bodies. We conclude that the peritrichous patterning of flagella of *B. subtilis* is different from the polar patterning of other bacteria, and we infer that the *B. subtilis* rod probes the PG for holes that can accommodate the machine.

## INTRODUCTION

Subcellular localization is an important mechanism to control the amount and function of protein complexes. In bacteria, the subcellular localization of proteins became widely recognized when TEM immunogold-staining showed that the cell division protein FtsZ localized to the division plane, and methyl-accepting chemotaxis proteins localized as clusters near cell poles (1–3). An example of subcellular localization in bacteria, the discovery of which predates the discovery of FtsZ localization by many decades, is the patterning of the bacterial flagellum. Bacteria have long been known to assemble a species-specific number of flagella ranging from one to many per cell, localized to poles or along the length of a rod-shaped body (peritrichous), and phylogenetic conservation suggests that both flagellar number and position are selectable (4–6). Precisely how bacteria arrive at their species-specific number and pattern of flagella is poorly understood, and the two phenomena may be related (7, 8). Flagella are complex, sequentially assembled trans-envelope nanomachines (9–11), and their patterning is likely determined early in synthesis.

**Fig 1:**
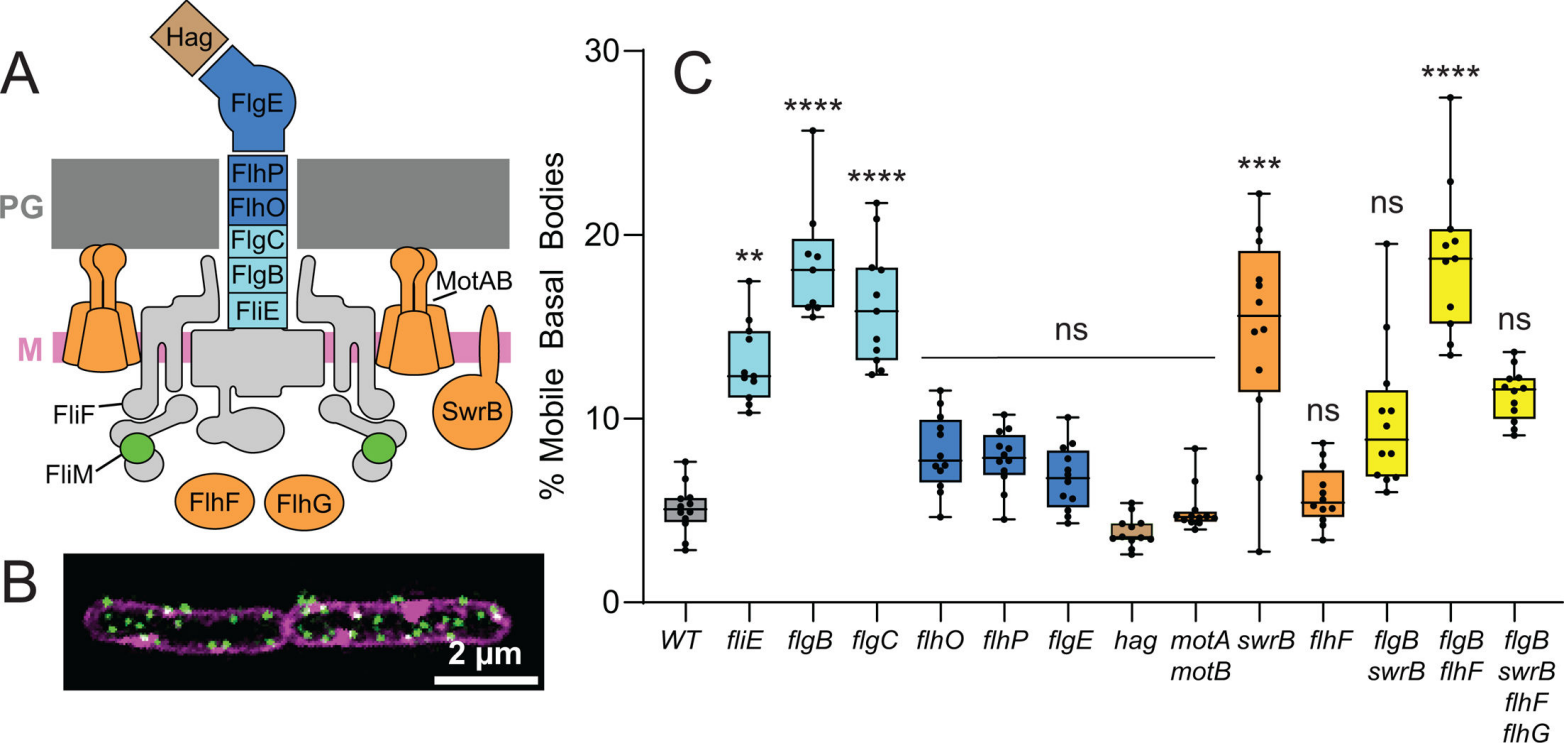
Flagellar basal bodies are immobilized by the proximal rod. (**A**) Cartoon cross-section of a flagellum, including the basal body and C-ring (light gray), the proximal rod (light blue), distal rod-hook (dark blue), filament (brown), and proteins that control rotation, assembly, and patterning (orange). (**B**) 3D SIM fluorescence micrograph of two conjoined *B. subtilis* cells with membrane false colored magenta and FliM-GFP puncta false colored green. Scale bar is 2 µm. (**C**) Timelapse TIRF microscopy was used to monitor flagellar basal bodies as indicated by fluorescent puncta of FliM-GFP. Images were captured every 250 ms for 1 min. Basal body puncta were counted, tracked, and classified as stationary or mobile based on analysis of their mean squared displacements (see Materials and Methods). Three to four movies were generated for each biological replicate and scored separately to form a point on the graph. Three biological replicates were made for each strain. Boxes represent the inner quartile range of the data points and are color-coded according to the corresponding subunits indicated on the cartoon at the right. The line through the boxes indicates the median value, and whiskers indicate the range of values measured for each data set. The following strains were used to generate the data: WT (DK1906), *fliE* (DK5081), *flgB* (DK5082), *flgC* (DK5268), *flhO* (DK5083), *flhP* (DK5084), flgE (DK4892), *hag* (DK4347), *motAB* (DB897), *swrB* (DK479), *flhF* (DK2118), *flgB swrB* (DB1637), *flgB flhF* (DK7871), and *flgB swrB flhF flhG* (DK9675).

Flagellar synthesis begins with the assembly of a transmembrane basal body composed of a type III secretion apparatus surrounded by the flagellar baseplate protein FliF (12–17). Next, a cytoplasmic ring of proteins (the C-ring) is assembled onto the membrane complex that forms a gear-like rotor and directional control system (18–21) (Fig. 1A). Once the C-ring is assembled and basal body formation is complete, the flagellar type III secretion system (FT3SS) becomes active and secretes subunits that polymerize atop the basal body to form the axle-like rod (12, 13, 19, 22, 23). The rod extends away from the cell and transits the peptidoglycan (PG) as well as the outer membrane, if present (19, 24) (Fig. 1A). The FT3SS also exports subunits of the universal joint-like hook, and later, subunits of the long helical filament, which when rotated, serves as a propeller (25, 26). Complex regulatory networks ensure that the genes that encode the flagellar structure are more or less expressed in the order that the products are assembled, and structural checkpoints control secretion order and timing (27, 28). How and when flagella become patterned during assembly is poorly understood for any bacterium, but patterning often relies on cytoplasmic regulators similar to those that control cell division site selection (7, 8).

The localization of proteins in general is thought to occur by one of two conceptual models: targeted assembly or diffusion-and-capture (29). During targeted assembly, patterning proteins determine the future position of a complex, which is then assembled at that location. For instance, transmembrane proteins at one pole of the cell catalyze basal body formation in bacteria with single polar flagella (30–33). Next, in gram-negative bacteria, the rod-cap chaperone is either fused to or associated with a PG lyase such that rod polymerization is thought to create holes in the PG of sufficient diameter to allow rod transit (34–37). Finally, bushing proteins are assembled as rings around the rod to allow free rotation in the context of the envelope and govern the transition from rod to hook assembly (38–40). Thus, some bacteria predetermine the basal body location, and rod-cap activity ensures that the flagellar rod, PG pores, and the P- and L-ring bushings are synthesized in register. During diffusion-and-capture, however, proteins and/or complexes are assembled randomly and then diffuse or otherwise move to their ultimate location, where they are captured by structural or biochemical components of the cell. Whether or not nascent flagella are mobile prior to completion, what cellular components are recognized by flagella, and how cytoplasmic flagellar patterning proteins would govern basal body capture is unclear.

The gram-positive bacterium *Bacillus subtilis* assembles approximately 25 flagellar basal bodies per cell peritrichously organized along the cell body in a non-random grid-like pattern, and it appears to lack critical components of gram-negative targeted assembly including the rod-cap, a dedicated PG lyase, bushing proteins, and transmembrane recruiters (28, 40–43). Here, we explore early patterning events in *B. subtilis* by studying basal body dynamics. In wild-type cells, basal bodies exhibit a negligible rate of subunit exchange, and while they are predominantly stationary, a small number of mobile basal bodies were observed. Using an inducible flagellar system, the fraction of mobile basal bodies was found to be high soon after induction, and this fraction decreased over time, consistent with a model in which mobility is a property of early-stage assembly and basal bodies become captured as they mature. Flagellar rod assembly is part of basal body capture, as mutation of early rod subunits increased the percentage of mobile basal bodies, and we infer that FlgC is the rod subunit most likely to make first contact with, and be restrained by, the PG. Finally, rod mutants also have basal body patterning defects similar to mutants defective in the cytoplasmic regulatory protein FlhF that governs flagellar patterning. How *B. subtilis* flagella transit the PG without a dedicated PG lyase to create holes for the rod is unclear, and how flagella are arranged in a grid-like pattern is poorly understood. Our data support a model in which nascent basal bodies diffuse in the membrane and metastable rod assembly continuously probes a pre-existing pattern imprinted on the PG itself.

## RESULTS

### Flagellar basal bodies are largely static, and their subunits do not dynamically exchange

To distinguish whether flagellar patterning was primarily controlled by targeted synthesis or diffusion-and-capture in *B. subtilis*, we set out to determine if and when basal bodies were mobile. To observe flagellar basal bodies, a FliM-GFP fusion protein was incorporated into the cytoplasmic ring (C-ring) such that the flagella appear as puncta in fluorescence microscopy (41). To determine if basal bodies are mobile, cells were imaged using time-lapse total internal reflection fluorescence (TIRF) and single-particle tracking. Approximately 5% of tracked basal bodies scored in the wild type were identified as mobile over the time course observed (Fig. 1C; Movie S1), and the low percentage of mobile basal bodies is consistent with the fact that flagellar basal bodies have been used as stationary fiducials for comparing the movement of other proteins (44). We conclude that while the basal bodies of *B. subtilis* were overwhelmingly stationary, a small subpopulation of mobile particles exists.

A low percentage of FliM-GFP puncta might indicate either that basal bodies are occasionally mobile or that C-rings might detach from basal bodies and move independently. To test for FliM co-localization with the basal body, cells were doubly labeled: FliM was fused to a HaloTag and the major transmembrane flagellar housing protein FliF was fused to mNeonGreen. Although the FliM-HaloTag was partially functional for supporting swarming motility in single copy, the FliF-mNeonGreen fusion was non-functional (Fig. S1A), the two fluorescent tags nonetheless co-localized when expressed in the same cell and stained with a Halo reactive dye (Fig. S1B). Moreover, rare puncta of FliM-Halo and FliF-mNeonGreen were observed to move in cells, and when they did, co-localization was maintained (Movies S2 to S4). We conclude that FliM and FliF co-localize as a complex, and thus, the presence of mobile puncta of FliM-GFP indicates a subpopulation of assembled and mobile basal bodies in wild-type cells.

**Fig 2:**
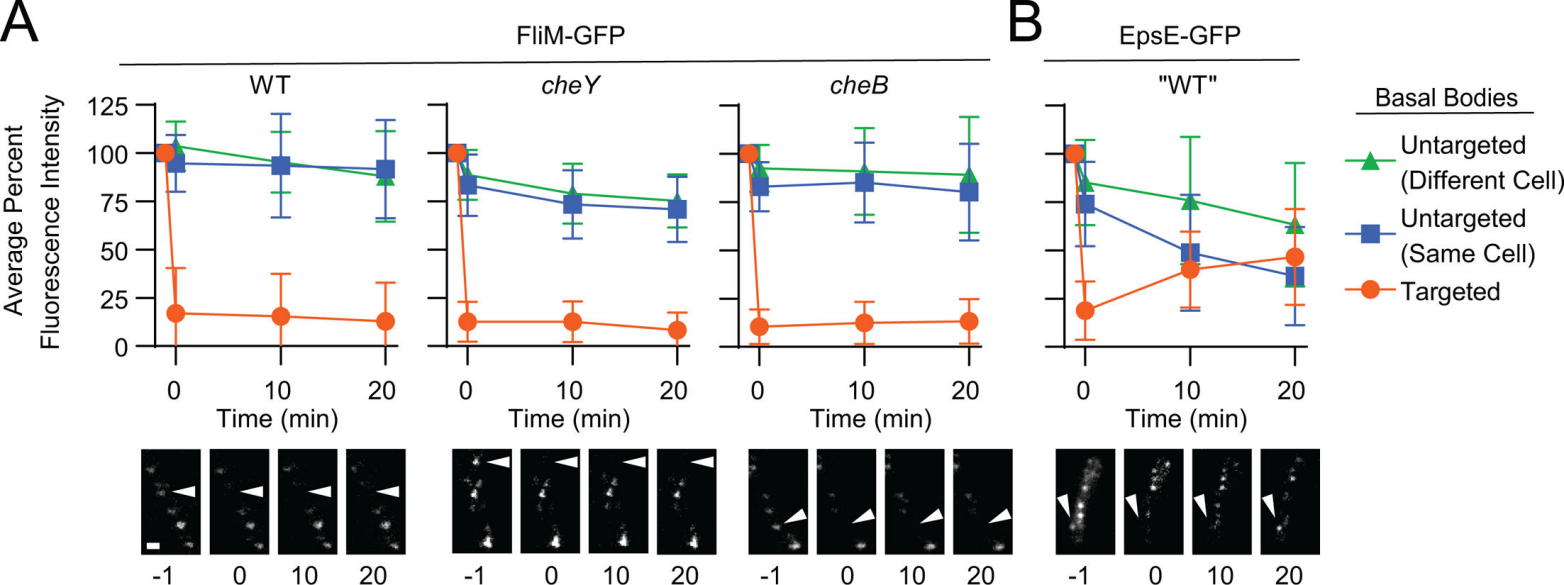
Flagellar C-ring subunits do not dynamically exchange between flagella. Cells expressing (**A**) FliM-GFP or (**B**) EpsE-GFP were imaged with TIRF microscopy before and after photobleaching in a variety of strains. After the first image was acquired, individual puncta were bleached of fluorescence via targeted laser excitation. For each strain, 10 puncta were targeted for photobleaching (orange circles), and fluorescence was monitored over time. In addition, fluorescence was monitored for 10 puncta that were in the same cell but not targeted (blue squares) as well as 10 puncta from an untargeted cell elsewhere in the same field (green triangle). Each data point represents the average % of original fluorescence intensity for 10 measurements across three biological replicates, and error bars are the standard deviation. The following FliM-GFP expressing strains were used to generate panel A: wild-type (DK1906), *cheY* (DK1977), and *cheB* (DK1991). For panel B, the “wild-type” strain (DS2955) was also mutated for the biofilm repressor SinR to express EpsE-GFP, and mutated for EpsH to abolish cell clumping that occurs in the absence of SinR (51, 52). Below each graph are representative micrographs of a single cell from each strain, with white arrows indicating the targeted basal body. Numbers indicate the time in minutes relative to laser bleaching. Scale bar is 1 µm.

C-rings are comprised of many subunits, and while whole C-ring assemblies did not appear to exchange between basal bodies, individual subunits of FliM have been shown to exhibit dynamic exchange between basal bodies in *Escherichia coli* (16, 45–50). As we are using FliM-GFP as a marker for *B. subtilis* basal body location and dynamic FliM exchange has been reported in *E. coli*, we set out to determine whether individual FliM subunits were dynamic in *B. subtilis* by fluorescence redistribution after photobleaching (FRAP). Briefly, puncta of FliM-GFP in the presence of wild-type FliF were bleached by exposure to localized high-intensity laser excitation, and fluorescence was measured at intervals by TIRF. Puncta in the laser-exposed zone of targeted cells experienced a dramatic decrease in fluorescence intensity, and fluorescence intensity did not recover over time (Fig. 2A). As a control, puncta outside the laser-exposed zone within a targeted cell experienced a minimal loss of fluorescence that was comparable to puncta in untargeted cells (Fig. 2A). We conclude that our FliM-GFP reporter of flagellar basal bodies does not appear to exhibit dynamic subunit exchange, at least in wild-type *B. subtilis* cells.

The rate of dynamic exchange of FliM subunits in the *E. coli* flagellar basal body was shown to be altered by mutation of the chemotaxis signaling system (46, 48, 49). Therefore, to determine if the seemingly stable C-rings of *B. subtilis* could be made dynamic under some conditions that could affect present and future interpretations with the reporter, FRAP experiments were performed on cells in which the C-ring was conformationally locked to cause exclusive rotation in either the clockwise or counterclockwise direction by mutation of either the CheB methylesterase or the CheY response regulator (53–56). Similar to observations of wild-type cells, FliM-GFP puncta in cells mutated for CheB or CheY exhibited little to no dynamic protein exchange of individual subunits, as neither fluorescence recovery of target basal bodies nor fluorescence loss of off-target basal bodies was observed after photobleaching (Fig. 2A). Thus, it appears that although the C-rings of *E. coli* dynamically exchange structural subunits, the C-rings of *B. subtilis* do not.

While structural subunits of the *B. subtilis* flagellar C-ring appeared static, non-structural regulatory proteins have been shown to interact with the C-ring under certain conditions. For instance, EpsE is a bifunctional glycosyltransferase and flagellar clutch that inhibits flagellar rotation by binding to and disconnecting the FliG rotor component of the C-ring from power-generating stators (51, 57–59). We were concerned that our lack of observed subunit exchange could have been due to technical reasons that masked any potential positive result. Thus, we sought to determine whether a non-structural protein like EpsE could experience dynamic exchange under the same conditions. Unlike FliM-GFP, photobleached puncta of EpsE-GFP recovered fluorescence over time, and as fluorescence recovered, off-target EpsE-GFP puncta in the same cell experienced commensurate fluorescence loss (Fig. 2B). We conclude that regulators of C-ring function, like EpsE, dynamically exchange under our conditions, but structural subunits of the C-ring itself do not. In sum, we conclude that FliM-GFP puncta remain associated with their respective basal body both as individual subunits and as full C-ring assemblies, and thus, the mobile dots we observe likely indicated the movement of entire basal bodies, and not as separate or partially formed C-rings.

### Flagellar basal bodies are mobile early in assembly

**Fig 3:**
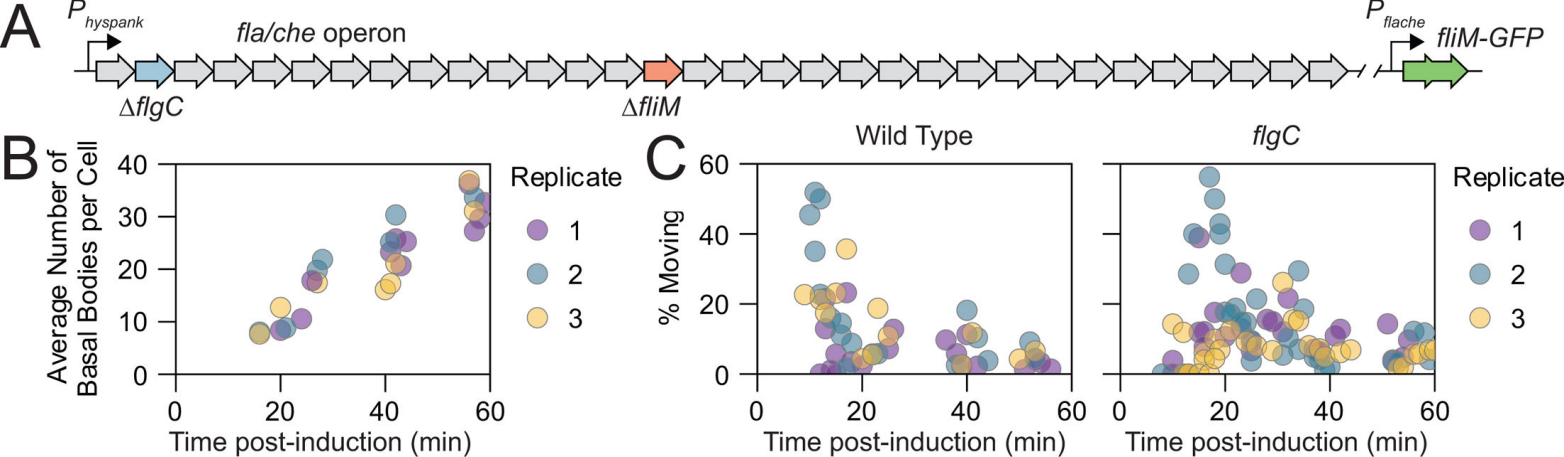
Flagellar basal bodies are mobile early in assembly. (**A**) Schematic showing genetic modifications made to strains used in this figure. For the wild-type strain DK31, the native *P_flache_* promoter of the *fla/che* operon was replaced with an IPTG-inducible *P_hyspank_* promoter. In addition, the *fliM* gene was deleted (orange), and ectopically reintroduced as *fliM-GFP* under the control of *P_flache_* (green). For the *flgC* mutant strain DB2199, a similar strain was generated in which the *flgC* gene (blue) was also deleted. (**B**) Wild-type DK31 cells described in panel A were imaged with 3D SIM at various time points after the addition of IPTG. Each bubble on the plot shows the average number of basal bodies per cell in a 3D-SIM image. (**C**) The wild-type DK31 (left) and *flgC* mutant (DB2199) were imaged with time-lapse TIRF microscopy at varying time points after the addition of IPTG. Images were captured every 250 ms for 1 min. Basal body puncta were counted, tracked, and classified as stationary or mobile based on analysis of their mean squared displacements. Three to four movies were generated for each biological replicate and scored separately to form a bubble on the plot.

While the majority of basal bodies were stationary, a minority were mobile, and we hypothesized that the mobile subpopulation might represent immature basal bodies at an early stage of assembly. To observe the behavior of immature basal bodies, a strain was created in which basal body formation was placed under the control of an IPTG-inducible promoter (41). Specifically, the native promoter of the 27 kb long *fla/che* operon was replaced by the IPTG-inducible *P_hyspank_* promoter. Moreover, the native *fliM* gene was deleted, and a FliM-GFP construct was expressed from the *P_flache_* promoter at an ectopic site (41) such that FliM-GFP would be present prior to IPTG induction, and puncta formation would be restricted by nucleation, rather than gene expression and fluorophore maturation (Fig. 3A). Thus, in the absence of the inducer, the strain should contain no flagellar basal bodies, and all basal bodies observed after inducer addition would be newly synthesized (Fig. 3A). Cells were monitored by two different approaches. In one approach, the total number of basal bodies was counted at indicated time points using OMX 3D structured illumination microscopy (SIM), which achieves high resolution in three dimensions for fluorescent signals. In a complementary approach, time-lapse TIRF microscopy was used to focus on a single plane closest to the coverslip to isolate and quantify basal body mobility by single-particle tracking. Taken together, the two approaches give the number of basal bodies per cell and the proportion of basal bodies that were mobile.

In the absence of inducer, cells grew as long chains, and FliM-GFP signal was diffuse in the cytoplasm, indicating that FliM-GFP was expressed, but the *fla/che* operon was not, and consequently, there were no basal bodies upon which FliM-GFP could nucleate (41, 52, 60, 61). Next, IPTG was added at mid-log phase, and cells were observed at various time points by OMX-3D SIM and TIRF microscopy in separate experiments. Basal body puncta were observed at the earliest time points tested, and consistent with temporal assembly, the number of puncta increased over time (Fig. 3B; Fig. S2). Meanwhile, the percentage of mobile basal bodies was highest at the earliest time points and decreased over time (Fig. 3C; Movie S5). We conclude that newly synthesized basal bodies exhibit the greatest mobility, and that the fraction of mobile basal bodies decreases as they mature. We infer that at least part of flagellar patterning involves the movement of immature flagellar basal bodies, which are captured at a location later in flagellar assembly.

### Basal bodies are immobilized by synthesis of the flagellar rod

To determine the mechanism by which flagellar basal bodies are captured and immobilized, FliM-GFP puncta dynamics were measured by TIRF time-lapse microscopy in a variety of mutants. The stage in flagellar assembly that follows the formation of the basal body is the secretion and polymerization of subunits that make up the flagellar rod and hook (12, 13, 19, 23, 62, 63). The flagellar rod-hook is comprised of six different proteins which are sequentially assembled from most proximal to distal in the following order: FliE, FlgB, FlgC, FlhO, FlhP, and FlgE (24, 64, 65) (Fig. 1A). Mutation of FliE and the proximal rod subunits FlgB and FlgC increased the fraction of mobile basal bodies significantly over wild type as statistically determined using Kruskal-Wallis with Dunn’s post hoc test (Fig. 1B). Mutation of the distal rod subunits FlhO and FlhP, and the hook subunit, FlgE, appeared to increase basal body mobility, but the increase was not significantly different from wild type. We note that Kruskal-Wallis is less sensitive/powerful than a more standard ANOVA test but was necessary because the ANOVA is inappropriate when comparing data expressed as percentages (66). Nonetheless, the apparent, but not significant, increase in basal body mobility in some rod-hook mutants may have biological underpinning due to the reported property of metastability in which the rod-hook assembles and disassembles until completion and transition to the flagellar filament, at which point the rod-hook is stable (65). Perhaps consistent with metastability being restricted to the rod-hook, mutation of the flagellar filament subunit protein Hag yielded a low percentage of basal bodies, similar to wild type and lower than any rod-hook mutant (Fig. 1B). We conclude that mutation of the proximal rod increases the fraction of mobile basal bodies to approximately 20% at steady state.

We infer that rod synthesis immobilizes basal bodies due to spatial constraints imposed by insertion through cell wall PG. We further infer that FlgC is the first rod subunit to be constrained by PG because it is the most distal subunit, which when mutated, significantly increased the fraction of mobile basal bodies. To study the role of proximal rod synthesis in early basal body assembly, a strain was constructed in which *flgC* was deleted within the *fla/che* operon under the control of an IPTG-inducible promoter, and basal body mobility was monitored by tracking FliM-GFP with TIRF microscopy. Mutation of FlgC produced a high percentage of mobile basal bodies soon after induction, similar to that observed in the wild type, but basal body mobility remained higher for a longer duration before gradually coming to an elevated steady-state level (Fig. 3C). We conclude that the incorporation of FlgC into the proximal rod is an important early event that both increases the fraction of, and decreases the time to, basal body immobilization.

While rod transit through PG appears to contribute substantially to immobilization of the basal body, we note that as many as 80% of the basal bodies disrupted for the proximal rod were stationary (Fig. 1B). To explore whether non-structural regulatory proteins also contributed to basal body mobility, we focused on regulators involved in flagellar function and assembly. MotA and MotB form the stator complex that binds to PG and revolves in interaction with the C-ring to power flagellar rotation (67–71). Mutants simultaneously disrupted for MotA and MotB did not increase basal body mobility above wild-type levels, consistent with the fact that the stators are a late-acting component and likely associate after flagella have been fully assembled and patterned (Fig. 1B). SwrB is a single-pass transmembrane protein with a cytoplasmic domain that promotes basal body maturation and activation of the FT3SS (23, 72). Mutation of SwrB significantly increased basal body mobility above that of wild-type resembling cells mutated for the early rod subunits, and simultaneous mutation of SwrB and the early rod subunit FlgB did not further increase basal body mobility (Fig. 1B). We conclude that SwrB likely immobilizes basal bodies by activating flagellar type III secretion and promoting proximal rod subunit export.

Another set of regulators tested was FlhF and FlhG, partner proteins that control flagellar patterning. We considered the possibility that these proteins may control patterning by altering the frequency of basal body mobility. FlhG is a MinD-like ATPase which, when mutated, abolishes distance control between basal bodies and causes basal body aggregation in the cell (41, 73–75). While mobile basal bodies were occasionally observed in *flhG* mutant cells (Movie S6), a percentage of mobile basal bodies could not be quantified as basal body aggregation confounded puncta counting. Qualitatively, however, it did not appear that the frequency of mobile basal bodies was higher in the *flhG* mutant than in the wild type. FlhF is an SRP-like GTPase thought to interact with FlhG, and mutation of FlhF causes an asymmetric flagellar distribution that often manifests as a concentration of flagella toward one cell pole (41, 73, 76, 77). A mutant disrupted for FlhF did not significantly increase basal body mobility above wild-type levels, and simultaneous mutation of FlhF and FlgB, or FlgB, SwrB, FlhF, and FlhG did not increase basal body mobility above mutation of FlgB alone (Fig. 1B). We conclude that FlhF and FlhG do not control patterning by regulating the frequency of mobile basal bodies. Thus, the flagellar rod appears to be the primary determinant of basal body immobilization, at least with respect to other probable candidates within the known flagellar assembly system.

### The flagellar rod is required for basal body patterning

**Fig 4:**
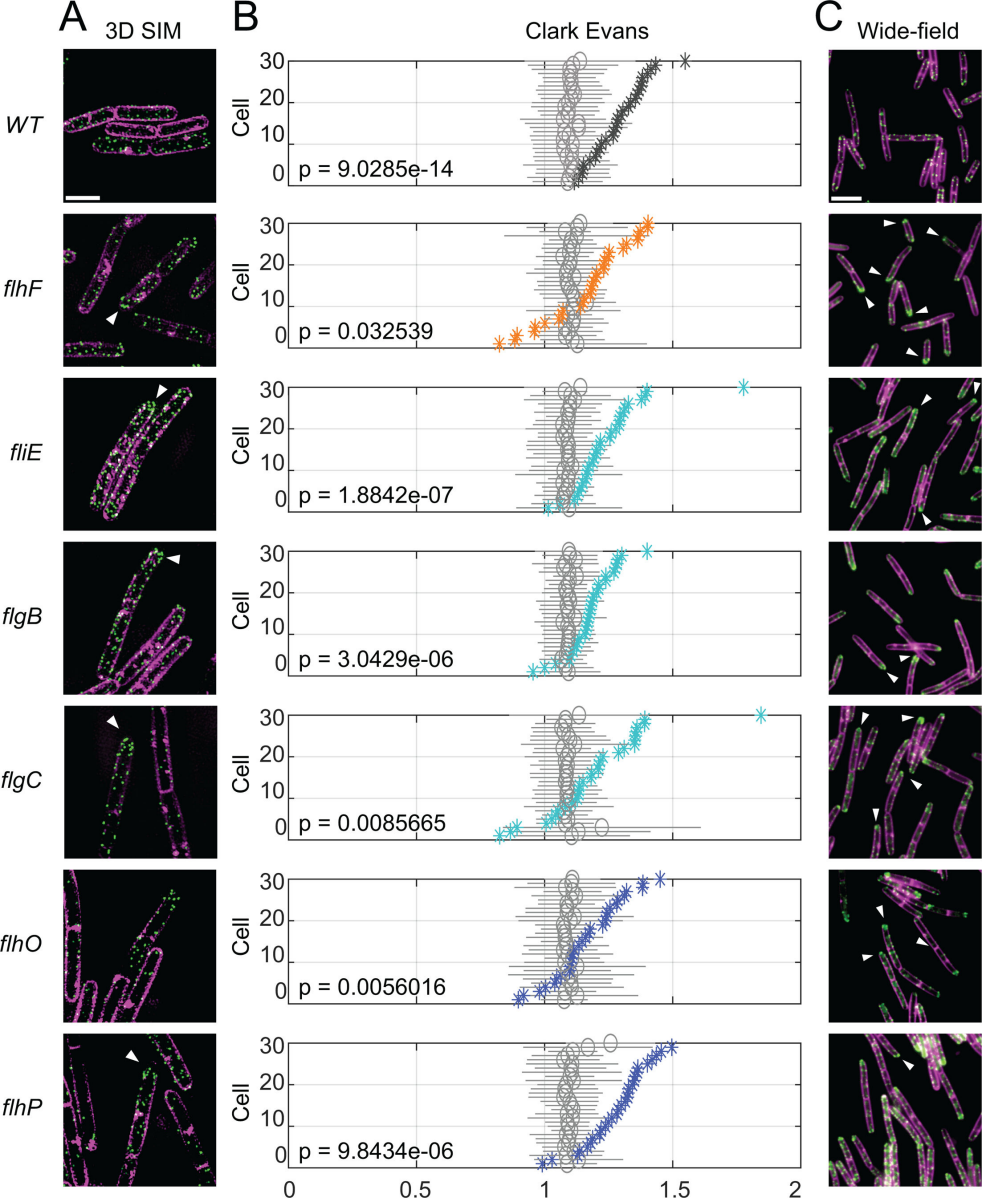
The flagellar rod is required for basal body patterning. (**A**) 3D SIM images of FliM-GFP (green) representing the flagellar basal body and FM4-64 (magenta) showing the cell membrane. Scale bar is 2 µm. (**B**) Clark-Evans distributions were calculated from the NNMD score from each cell (Fig. S4A), and the values are represented as a colored asterisk for each individual measured. Moreover, the same number of basal bodies within each individual cell was randomly redistributed and remeasured 1,000 times to generate a mean (gray circle) and standard deviation (gray bar) projected at the same height as the corresponding individual (see Materials and Methods). A Clark-Evans value of 1 indicates a random distribution of basal bodies, whereas Clark-Evans values of 0 and 2 indicate a non-random clumped or gridded distribution, respectively. Thus, each individual is compared to a randomized representative of itself, and *P* values were obtained by a two-sided *t*-test for all cells within the strain. (**C**) Wide field images of FliM-GFP (green) representing the flagellar basal body and FM4-64 (magenta) showing the cell membrane. White carats indicate polar accumulation of FliM-GFP. Scale bar is 5 µm. The following strains were used to generate the data: WT (DK1906), *flhF* (DK2118), *fliE* (DK5081), *flgB* (DK5082), *flgC* (DK5268), *flhO* (DK5083), and *flhP* (DK5084).

Mutations that disrupted the proximal flagellar rod increased basal body mobility, but mutations that disrupted the patterning regulators FlhF and FlhG did not. To further explore the relationship between mobility and patterning, each strain was imaged using OMX-3D SIM (Fig. 4A), and the nearest neighbor mean distance (NNMD) was determined and used to calculate a Clark-Evans ratio (41) (Fig. 4B). A Clark-Evans ratio varies between 0 and 2, where a value of 1 indicates a random distribution, and values near 0 or 2 indicate a non-random distribution with either clumped or grid-like quality, respectively (78). In wild type, each basal body is synthesized with an NNMD greater than that which would be predicted by chance (41), and the Clark-Evans ratio was between a value of 1 and 2, consistent with a grid-like distribution (Fig. 4B). A randomized data set was generated in which the same number of basal bodies within each cell were randomly repositioned and measured 1,000 times, taking into account the resolution limit of OMX, an adjustment, which on its own, resulted in a slight increase in the Clark-Evans ratio (Fig. 4B). Statistical comparison of the observed and randomized data sets supported previous observations that wild-type basal body distribution significantly differs from random and exhibits a grid-like character.

Mutation of FlhF resulted in a distribution of basal bodies that was more random than wild type with a statistical *P* value that barely satisfied the significance threshold of 0.05 when compared to the randomized dataset (Fig. 4B). Mutation FliE and each of the flagellar rod structural subunits FlgB, FlgC, FlhO, and FlhP, also indicated an increase in the randomness of basal body insertion which while statistically different from the randomized dataset, exhibited an elevated *P*-value relative to that of the wild type (Fig. 4B). Together, the basal body distributions of each mutant were statistically different from random, suggesting that a factor other than FlhF and the rod governs patterning potential, but the increase in *P* value may indicate that the rod nonetheless plays a role in interpreting or enforcing a pre-established pattern. An increase in randomness could lead to an asymmetric distribution of basal bodies over time as cells grow (79–81), and cells mutated for FlhF have been shown to accumulate basal bodies toward the poles (41) (Fig. 4C; Fig. S4B). Similarly, cells mutated for the flagellar rod also appeared to accumulate basal bodies toward the poles, particularly in the oldest poles located at the end of cell chains (Fig. 4C; Fig. S4B). We conclude that while basal body mobility and patterning are genetically separable, mutation of the extracytoplasmic flagellar rod subunits confers defects in patterning that are similar to defects observed in the cytoplasmic FlhF flagellar patterning protein. We further conclude that the rod both immobilizes basal bodies and participates in pattern formation.

## DISCUSSION

**Fig 5:**
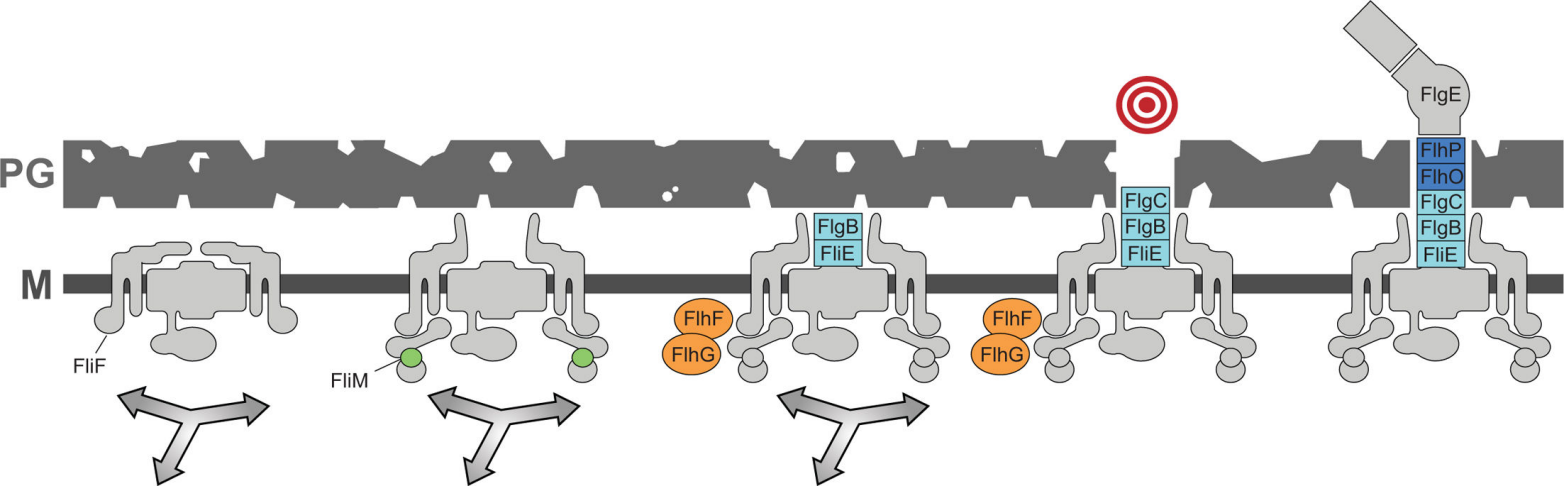
Basal bodies are immobilized by rod assembly. A model of the steps in dynamic flagellar assembly. Basal bodies are mobile (arrows) but inactive and become active for secretion by assembly of the C-ring (FliM, colored green) (23). Next, rod subunits are secreted and polymerized to form the proximal rod. If the local peptidoglycan (PG) superstructure does not sterically permit assembly of the full rod, rod metastability causes subunits to disassemble to a point below the steric hindrance of the PG and remobilize the basal body. Thus, the rod probes the PG for permissive holes, and if a hole is found of sufficient diameter, the rod-hook assembly will proceed to completion, the basal body will transition to filament subunit export and assembly, and lock the entire structure in place. The patterning proteins FlhF and FlhG (colored orange) interact with FliF and the C-ring, respectively, and either expedite basal body search or restrict which holes may be occupied, to create a symmetrically distributed, grid-like pattern of flagella.

The mechanism behind how flagella are positioned on the bacterial cell surface is poorly understood. Here, we explore the earliest stages of flagellar pattern acquisition in *B. subtilis*. We show that basal bodies are predominantly stationary at steady state but are mobile early in their synthesis using fluorescently labeled FliM as a proxy. As FliM loading and C-ring completion are required for basal body maturation and activation of the secretion system (21–23, 82, 83), tracking puncta of FliM allows observation of events throughout the assembly process. Mobile basal bodies become immobilized and patterned as they mature, and both patterning and immobilization depend on structural subunits of the flagellar rod as they transit the PG (Fig. 5). While the average pore size in PG is smaller than the diameter of the rod (21, 63, 84, 85), new observations and models of PG superstructure invoke heterogeneous porosity (86–89). We argue that basal bodies diffuse in the membrane until rod polymerization is permitted at a pore of sufficient diameter, thereby capturing the complex. Thus, flagella of *B. subtilis* seek holes in the wall rather than make them, and cytoplasmic patterning systems somehow restrict which holes may be occupied by the rod.

Our model incorporates the features of the flagellar rod that differentiate it from the hook and filament. First, the hook and the filament are primarily made of single proteins FlgE (65, 90, 91) and Hag (FliC) (92–94), respectively, but the rod is comprised of the basal body connector FliE (95, 96) and four different homologous structural subunits FlgB, FlgC, FlhO (FlgF), and FlhP (FlgG) (11, 65, 97). Rod subunits are secreted through the rod itself and sequentially stacked in helices of 6–24 subunits (11, 98), and our data suggest that FlgC, as the most distal subunit necessary to immobilize the basal body, is likely the first subunit to encounter PG. Second, the hook and filament are polymerized by dedicated cap chaperones (FlgD and FliD, respectively) (99, 100), and gram-negative bacteria encode a dedicated rod cap/PG lyase complex (FlgJ) thought to make holes for rod transit (34–36, 101). *B. subtilis,* however, seems to lack both a rod cap and a dedicated flagellar PG lyase, rendering the model in which the rod creates holes in the PG seemingly inapplicable (28, 42, 43). Third, whereas the hook and filament are stable structures, the rod is metastable, meaning it can both polymerize and depolymerize (a phenomenon observed primarily when completion is impaired) (12, 13, 19, 65). We speculate that if the rod is prevented from completion due to steric constraints imposed by the PG, rod assembly will stall and depolymerize until the basal body is remobilized and relocated for another attempt. In short, we suggest that stochastic rod assembly probes the PG superstructure. The flagellar secretion system exports approximately 10 subunits per second, so sampling and relocating could be rapid (102, 103).

The connection between rod assembly and basal body mobility is most clearly observed when flagella synthesis is artificially induced. Using an inducible system, a high percentage of basal bodies were mobile soon after induction, and this fraction decreased with time, consistent with a diffusion-and-capture model for flagellar patterning. After 40 minutes of induction, both the number of basal bodies and pattern are similar to that of the wild type at steady state (Fig. 3B; Fig. S2 and S3). Thus, we infer that whatever restricts basal body mobility and patterning is either encoded within the induced flagellar regulon itself and rapidly imprinted, or is exogenous to the flagellar regulon and pre-existent at the time of induction. The two possibilities are unified in our model, in which rod protein assembly searches for holes in a pre-existent pattern intrinsic to the PG, and flagellar rod mutants not only increase basal body mobility but display a patterning defect. Indeed, inducing flagellar synthesis in a *flgC* mutant prolonged the duration of elevated basal body mobility before coming to rest at the basal level.

While flagellar rod assembly immobilizes approximately 20% of basal bodies, ~80% remained immobile after rod ablation. Some basal bodies may appear immobile for technical reasons. Perhaps all basal body puncta are mobilized in a rod mutant, but some move too slowly to detect displacement within our experimental parameters, and we note that basal bodies in *E. coli* have been reported to move in a manner consistent with both free and restricted diffusion (104). We further note that membranes are heterogeneous and basal bodies are large multi-subunit complexes that could be stochastically distributed between regions of high and low membrane fluidity (105–107). Furthermore, experimental parameters such as agarose pad firmness or composition may affect mobility, and we found that mobility decreased with intense fluorescence excitation, as in the case where multiple fluorophores were simultaneously imaged over long time periods. Alternatively, rod-independent immobilization might be mediated by FliF, part of the flagellar secretion system, membrane lipid corralling proteins, or an as-yet-undiscovered protein. Whatever the case, the predominance of basal body immobility permitted pattern analysis, even when partially mobilized by rod mutation.

Basal bodies are patterned by the cytoplasmic proteins FlhF and FlhG, which when mutated disrupt pattern formation but not flagellar synthesis or motility. FlhF is thought to interact with the basal body protein FliF, and in *B. subtilis*, FlhF inhibits polar accumulation of flagella (41, 108, 109). FlhG is thought to interact with the C-ring proteins FliM and FliY in *B. subtilis,* and FlhG increases the basal body NNMD to inhibit basal body aggregation (41, 74, 110). We note three factors that confound statistically significant analysis of pattern distribution. First, as large numbers of basal bodies accumulate toward one pole in cells mutated for FlhF or aggregate in foci in cells mutated for FlhG, their proximity may fall below the limit of resolution, resulting in both a failure to obtain accurate basal body numbers and distances. Second, while *B. subtilis* produces a rather large number of flagella with respect to other bacteria, the number is still small enough such that minor changes on either side of the midcell yield high variation in asymmetry values, further confounded by the counting problem mentioned previously. Finally, polar flagellar accumulation (in the absence of FlhF) and flagellar aggregate size (in the absence of FlhG) appear particularly dramatic in a subpopulation, and thus inequal inheritance can produce a wide variance that is lost when averaged over the entire population.

Despite difficulties in defining some aspects of flagellar patterning, here we show that mutation of the flagellar rod resembles mutation of FlhF for basal body distribution, with a qualitative accumulation of flagella toward old cell poles. Why mutations of intracellular regulators and extracellular structural subunits have similar patterning defects is unclear but supports a dual sensory/regulatory role for rod assembly. We infer that basal body mobility, as indicated by the assembly state of the rod, could be transmitted to the cytoplasm through conformational changes in the basal body with which FlhF and FlhG interact. In turn, FlhF and FlhG could somehow restrict rod formation to a subset of permissible holes in the PG based on basal body proximity. Ultimately, we suggest that a grid resides in the architecture of the PG itself and that rod assembly and FlhF/FlhG activity cooperate to interpret the grid. The grid then is a consequence of cellular PG synthesis, with basal bodies searching for permissible pores, but precisely how the processes combine to form a grid-like symmetrical distribution of mature flagella remains unknown.

## MATERIALS AND METHODS

### Strains and growth conditions

*B. subtilis* strains were grown in lysogeny broth (LB) (10 g tryptone, 5 g yeast extract, and 5 g NaCl per L) broth or on LB plates fortified with 1.5% Bacto agar at 37°C. As indicated (see “Preparation of cells for microscopy”), cells were resuspended in casein hydrolysate (CH) media, prepared as described previously (111). When appropriate, antibiotics were included at the following concentrations: 10 µg/mL tetracycline, 100 µg/mL spectinomycin, 5 µg/mL chloramphenicol, 5 µg/mL kanamycin, and 1 µg/mL erythromycin plus 25 µg/mL lincomycin (*mls*). Isopropyl β-d-thiogalactopyranoside (IPTG, Sigma) was added to the medium at the indicated concentration when appropriate.

### Strain construction

All PCR products were amplified from *B. subtilis* chromosomal DNA, from the indicated strains. All constructs were either transformed into an NCIB3610-derived natural competent strain DK1042 (112), or transformed into domesticated strain PY79 and then moved to the 3610 background using SPP1-mediated generalized phage transduction (113). Briefly, SPP1-mediated transduction was performed by generating a plaque lysate on *B. subtilis* grown in TY soft agar (1% tryptone, 0.5% yeast extract, 0.5% NaCl, 10 mM MgSO_4_, and 1 mM MnSO4, 0.5% Bacto agar). Recipient strains were grown to stationary phase in TY, 1 mL was diluted into 9 mL TY, and 25 µL lysate was added, followed by incubation at room temperature for 30 min and then selection on the respective antibiotic at 37°C overnight. For transductions in which spectinomycin, kanamycin, or chloramphenicol resistance was selected, 10 mM sodium citrate was added to the selection plate. All strains used in this study are listed in Table 1. All primers used in this study are listed in Table S2.

**TABLE 1:** Strains

Strain	Genotype
DB814	*ycgO::P_fla/che_-fliF cat*
DB897	Δ*fliM motAB::tet amyE::P_fla/che_-fliM-GFP spec comI^Q12L^*
DB1356	Δ*fliF* Δ*fliM amyE::P_fla/che_-fliM-halo spec ycgO::P_fla/che_-fliF-mNeongreen cat*
DB1359	Δ*fliM* Δ*fliE amyE::P_fla/che_ fliM-GFP spec swrB::tet*
DB1394	Δ*fliF ycgO::P_fla/che_-fliF-mNeongreen cat*
DB1413	Δ*flgB-fliF* Δ*fliM amyE::P_fla/che_-fliM-halo spec ycgO::P_fla/che_-fliF-mNeongreen cat*
DB1673	Δ*flgB swrB::tet* Δ*fliM amyE::P_fla/che_-fliM-GFP spec*
DB2199	*comI^Q12L^ P_fla/che_* Ω *P_hyspank_ -fla/che operon kan* Δ*flgC* Δ*fliM amyE:: P_fla/che_-fliM-GFP spec*
DK31	*P_fla/che_*Ω*P_hyspank-_fla/che operon kan* Δ*fliM amyE::P_fla/che_-fliM-GFP spec*
DK479	*swrB::tet* Δ*fliM amyE::P_fla/che_-fliM-GFP spec comI^Q12L^*
DK1042	*comI^Q12L^*
DK1906	Δ*fliM amyE::P_fla/che_-fliM-GFP spec comI^Q12L^*
DK1977	Δ*fliM amyE::P_fla/che_-fliM-GFP spec* Δ*cheY comI^Q12L^*
DK1991	Δ*fliM amyE::P_fla/che_-fliM-GFP spec* Δ*cheB comI^Q12L^*
DK2117	Δ*flhG* Δ*fliM amyE::P_fla/che_-fliM-GFP spec comI^Q12L^*
DK2118	Δ*flhF* Δ*fliM amyE::P_fla/che_-fliM-GFP spec comI^Q12L^*
DK4347	Δ*hag* Δ*fliM amyE::P_fla/che_-fliM-GFP spec*
DK4892	Δ*flgE* Δ*fliM amyE::P_fla/che_-fliM-GFP spec*
DK5081	Δ*fliE* Δ*fliM amyE::P_fla/che_-fliM-GFP spec*
DK5082	Δ*flgB* Δ*fliM amyE::P_fla/che_-fliM-GFP spec*
DK5083	Δ*flhO* Δ*fliM amyE::P_fla/che_-fliM-GFP spec*
DK5084	Δ*flhP* Δ*fliM amyE::P_fla/che_-fliM-GFP spec*
DK5268	Δ*flgC* Δ*fliM amyE::P_fla/che_-fliM-GFP spec comI^Q12L^*
DK7871	Δ*flgB* Δ*flhF* Δ*fliM amyE::P_fla/che_-fliM-GFP spec*
DK9636	Δ*fliM amyE::P_fla/che_-fliM-halo spec*
DK9675	Δ*flgB* Δ*flhFflhG swrB::kan* Δ*fliM amyE::P_fla/che_-fliM-GFP spec*
DS2955	Δ*epsE sinR::kan epsH::tet thrC::P_epsA_-epsE-GFP mls*

To generate the translational fusion of FliF to mNeonGreen, the region containing the 5′ end of *ycgO*, the *P_flache_* promoter, and *fliF* was amplified from strain DB814 using primer pair 7620/8303, and the region containing the mNeongreen gene, the *cat* gene conferring chloramphenicol resistance and the 3′ end of *ycgO* was amplified from strain DB1341 using primer pair 8307/7621. The two fragments were fused by Gibson assembly and transformed into *B. subtilis*.

To generate pCD9 in which *fliM* was expressed from the *P_flache_* promoter with a convenient site for cloning 3′ end fusions, the *P_flache_-fliM* construct was amplified from pSG49 (41) using primer pair 8518/8519, digested with SphI and NotI, and cloned into the SphI-NotI sites of pAH25 encoding a spectinomycin resistance cassette between two arms of the *amyE* gene (a generous gift from Amy Camp, Mount Holyoke College).

To generate pDP580, which encodes a translational fusion of FliM to HaloTag, the gene encoding HaloTag was PCR amplified from an amplicon (generous gift of Malcolm Winkler, Indiana University) using primers 7681/7682, digested with NheI and NotI, and cloned into the NheI-NotI sites of pCD9.

### Swarm expansion assay

For the swarm expansion assay, cells were grown to mid-log phase at 37°C in LB broth, pelleted, and resuspended to 10 OD_600_ in pH 8.0 phosphate-buffered saline (PBS) (137 mM NaCl, 2.7 mM KCl, 10 mM Na_2_HPO_4_, and 2 mM KH_2_PO_4_) containing 0.5% India ink. Freshly prepared LB containing 0.7% Bacto agar (25 mL/plate) was dried for 10 min in a laminar flow hood, centrally inoculated with 10 µL of the cell suspension, dried for another 10 min, and incubated at 37°C. The India ink demarks the origin of the colony, and the swarm radius was measured relative to the origin. For consistency, an axis was drawn on the back of the plate, and swarm radii measurements were taken along this transect. Swarm expansion was measured every 30 min until wild type reached a radius of 30 mm (55).

### Preparation of cells for microscopy

Cells were grown in LB broth at 37°C until log phase (OD_600_ 0.5–0.9). For HaloTag microscopy, cells were incubated in LB broth at 37°C with 1 nM Janelia Fluor 549 for 10 min. For IPTG induction experiments, 1 mM IPTG was added to growing cultures at the early-to-mid log phase (OD_600_ 0.2–0.5) and cells continued to grow in its presence for 5, 10, 15, 30, or 45 min. A total of 1 mL of cells was then pelleted. For 3D SIM microscopy, cells were resuspended in 30 µL 5 µg/mL FM4-64 (Molecular Probes) and incubated at room temperature for 3 min to stain the membrane before being pelleted again. Cells were then washed with 1 mL water and pelleted again. All samples were resuspended in 30 µL water, then observed by spotting 4 µL of this suspension on a 1% agarose pad made with water. For IPTG induction experiments, 1 mM IPTG was also added to the agarose pad. For FRAP microscopy, which required longer time points, water was replaced by the defined rich CH media in all instances. CH media (114) is capable of sustaining growth while maintaining low background fluorescence during imaging.

### OMX microscopy

A GE Deltavision OMX 3D-SIM Super Resolution System V3.0 (Applied Precision) was used for FRAP experiments, TIRF microscopy, and 3D SIM. Images were acquired with the PCO.edge front illuminated sCMOS camera (PCO-Tech, Wilmington, DE). Image acquisition was directed by AcquireSR 4.5 (Applied Precision, Cytiva). For TIRF and FRAP, a 1.49 NA Apo N 60× oil objective was used with 1.514 refractive index oil. For 3D SIM, a 1.42 NA PlanApo 60× oil objective was used with 1.520 refractive index oil. Two channel images were acquired sequentially using the 568 (609–654 emission filter) and 488 (500–550 emission filter) nm lasers (Toptica, Model: iCHROME-MLE-LFK-GE). Single-channel images were captured using the 488 nm laser alone. Exposure times were 15–30 ms. 25–30% of the original 100 mW (488 nm) or 65 mW (568 nm) laser power was used for image acquisition. For time-lapse TIRF, images were taken every 250 ms for 1 min. Supplemental movies are played back at 60 frames per second. For 3D SIM, image reconstructions were made with the OMX-specific softWoRx 7.2.2 suite (DeltaVision).

### Basal body counting from 3D SIM images

To determine the average number of basal bodies per cell, 3D SIM images were first imported into IMARIS Microscopy Image Analysis Software. Individual cells were identified on the basis of FM4-64 membrane staining. FliM-GFP was used as a fiducial for basal bodies. To avoid bias in cell selection, all cells in one image were analyzed unless bleaching or poor focus rendered the cell outline too obscure to accurately determine cell endpoints or to differentiate between two cells. Cell selection began at the top left of the image and moved toward the bottom right until the set number of cells for that replicate were analyzed.

Counting of basal bodies in IPTG induction experiments was complicated by the fact that synthesis and assembly of basal bodies continue after cells are removed from media and taken to the microscope. As such, small variations in the time required to focus the microscope as well as the time required to acquire each sequential field could potentially lead to apparent variability in the number of basal bodies at each time point. Thus, the total time from the addition of IPTG to the acquisition of each individual image was recorded. In order to report accurate basal body numbers throughout *fla/che* induction, 50 cells for each of five incubation time points (see preparation of cells for microscopy) were counted, and the average number of basal bodies/time point was reported for the total time elapsed post IPTG induction for each field. This was repeated for three replicates for a total of 150 cells per time point, and therefore 750 cells within the experiment.

### Basal body distribution analysis from 3D SIM images

To avoid bias in cell selection, all cells in one image were analyzed unless bleaching or poor focus rendered the cell outline too obscure to accurately determine cell endpoints or to differentiate between two cells. Cell selection began at the top left of the image and moved toward the bottom right until the set number of cells for that replicate were analyzed. For spatial analysis of basal bodies (Fig. 4B), 10 cells were chosen for analysis in each of three replicates, consistent with the amount analyzed in a previous publication (41).

Assessing basal body spatial distribution requires distance measurements between points around the surface of a 3D bacterial cell with reference to the cell’s mid-point and ends. The 3D SIM images have a lateral resolution of >100 nm and axial resolution >200 nm. Basal body *X*, *Y*, and *Z* positions were determined in IMARIS by fitting to the fluorescent objects after entering information about the imaging conditions and microscope parameters. The *X* and *Y* positions of cell ends were selected by hand, using membrane staining as a reference for cell axes. Basal body and cell end positions were exported in calibrated micrometers to Excel spreadsheets.

Basal body positions were imported into MATLAB and associated with matrices for *X*, *Y*, and *Z* positions and for cell ends. Estimates of basal body distribution on the bacterial cell perimeter were created using the assumption that cells are roughly cylindrical. Each cell was centered by subtracting the mean *X*, *Y*, and *Z* basal body position for each respective dimension. Cells were rotated to have the long axis on *X* by creating a definite positive 3 × 3 matrix from the centered *X*/*Y*/*Z* positions and using singular value decomposition to create a rotation matrix. Because basal body positions were initially used to center the cell, the end-wall positions were applied to recenter the cell’s long axis to the cell’s midpoint. The basal body asymmetry over the long axis was determined as the ratio of higher to lower basal body count relative to the cell midpoint. Mean basal body distribution was determined by normalizing cell lengths and creating a histogram of 21 equal bins for relative basal body position with cells randomized for orientation (i.e., no preference for basal body asymmetry).

Basal body displacement was determined essentially as in Guttenplan et al. (41). A cylinder was created by fitting a circle to *Y*/*Z* projected basal body positions and then extending the 3D cylinder over the *X* axis. Nearest neighbor distance was calculated using the projected position of each basal body on the “unrolled” cylinder. Spatial distribution bias was estimated using a Clark-Evans formulation (78) with the cylinder surface (exclusive of cell ends) used for area estimation when calculating 2D density. The spatial bias estimation is a ratio of mean nearest neighbor distance and an assumed random (Poisson) 2D distribution over the same surface area, yielding a range from 0 (clumped) to 2 (maximally dispersed), with 1 representing a random distribution (78). Cell volume was calculated as the volume of the cylinder.

Clark Evans values approached a value of one for a random 2D distribution (i.e., uniform random on *X* and *Y*), where values less than 1 indicate clustering. CE values greater than 1 (0 < *R* < 2.149) infer gridding or non-random dispersion. The use of mean nearest neighbor distance for evaluating 2D spatial distributions has several limitations. For a rectangular area, the CE values for randomized data trend >1.0 owing to the inability to reach the nearest neighbor across an edge. This is not the case for the basal body data in one of two dimensions. Second, the expected variance for CE scales with density, meaning that bacterial strains with fewer or more variant basal body numbers should have more variation in CE values. And importantly, the expectation that the mean nearest neighbor distance for a random distribution should equal ½ (sqrt(density)) assumes that the measured distances do not have a resolution limit on the same order of magnitude as the mean nearest neighbor distance. In this case, the SIM resolution limit precludes measurements below ~100 nm, where the mean nearest neighbor distance may be 300–500 nm, depending upon density.

To estimate the robustness of the CE metric for indicating a deviation from ideally random basal body positioning and for comparisons between bacterial strains, we created an empirical approach simulating a random 3D distribution of basal body positions for each cell and applying the same CE determination methods (i.e., unrolling). To conservatively capture the relevant error sources, 1,000 simulated cells were generated for each measured cell using the basal body number and cell length per cell as input. To capture the error in determining cell diameter associated with basal body projection onto a cylinder, the radial distance from a central longitudinal axis was randomized using the mean radial distance of cell’s basal bodies ± the standard deviation. Therein, each simulated radius varied as a Gaussian function of the variation in fitting a cylinder to the 3D basal body positions, producing a range of cell surface areas related to basal body density. The basal body positions were subsequently generated by randomizing positions on *X* and the angle around the longitudinal *XY* axis to produce 3D *XYZ* positions. To account for finite microscope resolution, simulated cells were rejected until all nearest neighbor distances were greater than a 100 nm cutoff.

The CE metric for each cell was compared to 1,000 simulations of that cell. Plots of mean ± std CE from randomized positions showed the expected value above one and strain-dependent differences in standard deviation related to basal body density. Using the distribution of simulation CE values to create an empirical cumulative distribution function, we determined with what probability the measured CE value would be expected to appear in a population of cells with randomly distributed basal bodies.

### Fluorescent redistribution experiments

For FRAP experiments, cells were imaged immediately before and after the bleaching event, and for the 10- and 20-min time points using the TIRF settings described in OMX microscopy section. For the bleaching event, regions of cells were exposed to a 488 nm laser moving in a Lissajous pattern for a duration of 0.4 s. Ten cells per replicate were analyzed for a total of 30 cells per strain. Thirty cells were chosen as three times the total number analyzed in comparable work done in *E. coli* (46).

Cells were preferentially counted if the laser bleached only a small subset of the fluorescent puncta.

For each biological replicate within each strain, 10 regions of interest (ROIs) were drawn randomly on the field background, where no cells were present, and at each time point, fluorescence intensity was measured to give a background value. Next, ROIs were drawn around ten bleached puncta (targeted), 10 unbleached puncta in the same cells as the previous 10 (untargeted same-cell), and 10 unbleached puncta in cells with no localized bleaching (untargeted separate-cell). The fluorescence intensity of each punctum was measured at each time point, and the background at the given time point was subtracted. These values were then averaged and expressed as a percentage relative to the original average fluorescence intensity.

### Basal body tracking and mobility quantification

Movies of basal bodies with fluorescently labeled FliM-GFP subunits were analyzed with Palmari, a particle tracking plugin for Napari (115). Prior to analysis, movies were reviewed, and those with substantial drift or cell movement that would affect particle tracking were excluded. After analysis, movies in which only one punctum was detected were also discarded. At least three movies were considered per strain or condition. A mask determined by an intensity threshold on the time average of the entire movie was used to exclude regions without cells from analysis. Particles were detected by applying a Laplacian of Gaussian filter to each frame and identifying particles with intensities greater than one standard deviation above the mean intensity for that frame (116). For *fla/che* induction experiments, the intensity threshold was adjusted to account for the broader variance in particle densities. The centroid position of each particle was estimated using radial symmetry optimization (117). Particle localizations were linked into tracks using a simple tracking algorithm that links particles detected in consecutive frames within a 3-pixel search radius (118).

To quantify the mobility of labeled basal bodies, we considered tracks with localizations in at least 20 consecutive frames. Tracks were analyzed in 20-frame segments on a rolling basis. Mean squared displacements, $\langle r^{2}\rangle$, for all temporal lags, τ, within a segment were used to estimate the apparent diffusion coefficient, $D_{app}$. For Brownian motion, $\langle r^{2}\rangle (\tau )$ is proportional to $D_{app}$:


$$\left\langle r^{2}\right\rangle \left(\tau \right)=4D_{app}\tau +4\sigma^{2}$$

where τ is an integer multiple, i, of the 250 ms time interval between frames. $D_{app}$ was estimated using an analytical expression from weighted least squares optimization (119):


$$D_{app}=\frac{1}{4}\left(\frac{\sum_{i}n_{i}\sum_{i}n_{i}\tau_{i}{\left\langle r^{2}\right\rangle }_{i}-\sum_{i}n_{i}\tau_{i}\sum_{i}n_{i}{\left\langle r^{2}\right\rangle }_{i}}{\sum_{i}n_{i}\sum_{i}n_{i}\tau_{i}^{2}-{\left(\sum_{i}n_{i}\tau_{i}\right)}^{2}}\right)$$

Here, $n_{i}$ is the number of displacements used to calculate the mean squared displacement, ${\langle r^{2}\rangle }_{i}$, for a temporal lag, $\tau_{i}$.

We determined that 0.0003 µm^2^/s was the lower limit of detectable $D_{app}$ based on the localization uncertainty of these experiments. Particles that were considered mobile if their apparent diffusion coefficient exceeded 0.0003 µm^2^/s in at least 10% of segments.

Analysis was completed using a series of IPython notebooks that have been made available in a Github repository: https://github.com/BiteenMatlab/basal_body_tracking.

## SUPPLEMENTAL MATERIAL

Supplemental material mbio.00530-25-s0001.pdf Tables S1 and S2, Fig. S1 to S4, and movie legends.

Movie S1 mbio.00530-25-s0002.avi Rare basal body mobility is observed in wild-type cells.

Movie S2 mbio.00530-25-s0003.avi FliM-HaloTag and FliF-mNeongreen merged time lapse.

Movie S3 mbio.00530-25-s0004.avi FliF-mNeongreen channel only time lapse.

Movie S4 mbio.00530-25-s0005.avi FliM-HaloTag channel only time lapse.

Movie S5 mbio.00530-25-s0006.avi Mobile basal bodies observed 15 minutes after induction of the flache operon.

Movie S6 mbio.00530-25-s0007.avi Basal body in an flhG mutant is initially mobile and comes to a stop.

ASM does not own the copyrights to Supplemental Material that may be linked to, or accessed through, an article. The authors have granted ASM a non-exclusive, world-wide license to publish the Supplemental Material files. Please contact the corresponding author directly for reuse.
