# Supplementary material for: Nascent flagellar basal bodies are immobilized by rod assembly in Bacillus subtilis
Source: mBio. 2025 May 21;16(6):e00530-25. doi: 10.1128/mbio.00530-25 (PMC12153297; doi:10.1128/mbio.00530-25)
Supplement: Supplemental material — Tables S1 and S2, Fig. S1 to S4, and movie legends. [file mbio.00530-25-s0001.pdf]

SUPPLEMENTAL MATERIAL

Table S1: Statistical significance between basal body mobility frequencies of various mutants[a]

	WT	*fliE*	*flgB*	*flgC*	*flhO*	*flhP*	*flgE*	*hag*	*motA motB*	*swrB*	*flhF*	*flgB swrB*	*flgB flhF*	*flgB swrB flhF flhG*
**WT**														
fliE	.0088 **													
flgB	<0.0001 ****	>0.9999 ns												
flgC	0.0001 ***	>0.9999 ns	>0.9999 ns											
flhO	>0.9999 ns	>0.9999 ns	0.0761 ns	0.2573 ns										
flhP	>0.9999 ns	>0.9999 ns	0.0472 *	0.1624 ns	>0.9999 ns									
flgE	>0.9999 ns	0.4413 ns	0.0040 **	0.0148 *	>0.9999 ns	>0.9999 ns								
hag	>0.9999 ns	0.0001 ***	<0.0001 ****	<0.0001 ****	0.4318 ns	0.6649 ns	>0.9999 ns							
motA motB	>0.9999 ns	0.0054 **	<0.0001 ****	<0.0001 ****	>0.9999 ns	>0.9999 ns	>0.9999 ns	>0.9999 ns						
swrB	0.0025 **	>0.9999 ns	>0.9999 ns	>0.9999 ns	>0.9999 ns	>0.9999 ns	0.1850 ns	<0.0001 ****	0.0015 **					
flhF	>0.9999 ns	0.0543 ns	0.0003 ***	0.0011 **	>0.9999 ns	>0.9999 ns	>0.9999 ns	>0.9999 ns	>0.9999 ns	0.0185 *				
flgB swrB	0.8814 ns	>0.9999 ns	0.7514 ns	>0.9999 ns	>0.9999 ns	>0.9999 ns	>0.9999 ns	0.0315 *	0.6169 ns	>0.9999 ns	>0.9999 ns			
flgB flhF	<0.0001 ****	>0.9999 ns	>0.9999 ns	>0.9999 ns	0.0360 *	0.0212 *	0.0014 **	<0.0001 ****	<0.0001 ****	>0.9999 ns	<0.0001 ****	0.4579 ns		
flgB swrB flhF flhG	0.0723 ns	>0.9999 ns	>0.9999 ns	>0.9999 ns	>0.9999 ns	>0.9999 ns	>0.9999 ns	0.0013 **	0.0465 *	>0.9999 ns	0.3643 ns	>0.9999 ns	>0.9999 ns	

[A]Values of Kruskal-Wallis statistical significance with Dunn's post-hoc test for all pairwise comparisons of various mutants presented in Figure 1B. Boxes are colored gray for datasets that are significantly different from one another.

Table S2: Primers

Primer	Sequence
7620	GGCATCATTCACTGTATCGCTG
7621	GAGTGCTACACCTGTTATTGCAG
7681	AGGAGGCTAGCGGTTCCGCTGGCTCCGCTGC
7682	CTCCTGCGGCCGCCCGGTTAACCAGAAATTTCCAATGTAG
8303	TCCGGAACCGCTAGCGCCAGAACCAGCAGCGGAGCCAGCGGATCCATCCTCGGCCAGCCAGCT
8307	GGATCCGCTGGCTCCGCTGCTGGTTCTGGCGCTAGCGGTTCCGGAGTTTCGAAAGGAGAGGAGGAT
8518	AGGAGGTCGACAGGTACTTATATCAAGGTACTAAACAA
8519	GCGGCCGCAGAACCTGATCCAGATGCACCTCCGGAACCGCTAGCGCC

SUPPLEMENTAL FIGURE LEGENDS

Fig S1: Fluorescent fusions to FliM and FliF cause swarming defects. A) A quantitative swarm assay is shown in which each line is the average of three replicates. Circles represent wild type cells (DK1042). Squares represent cells expressing FilM-Halo as the only copy of FliM in the cell (DK9636). Triangles represent cells expressing FliF-mNeongreen as the only copy of FliF in the cell (DB1394). Inverted triangles represent cells expressing both FliM-Halo and FliF-mNeongreen (DB1356). **B)** 3D SIM images of wild type cells (DB1356) expressing FliM-Nalo (magenta, first column) and FliF-mNeongreen (green, second column).

Fig S2: Basal bodies increase in number throughout induction of the *fla/che* operon. A strain in which the P_{flache} promoter was replaced by the IPTG-inducible $P_{hyspank}$ promoter (DK31) was induced and imaged over time. Micrographs show FliM-GFP false-colored green, and FM4-64 membrane stain false-colored magenta. The first image shows cells in absence of inducer and the subsequent images show cells at the indicated times post induction.

Fig S3: Patterning of induced flagella is like that of the wild type. A strain in which the P_{flache} promoter was replaced by the IPTG-inducible $P_{hyspank}$ promoter (DK31) was induced for 41 minutes with 1 mM IPTG and basal body patterning parameters were measured. Left, fluorescence micrograph in which membrane was false-colored magneta, and FliM-GFP puncta false-colored green. Right, Clark-Evans distributions were calculated from the NNMD score from each cell, and the values are represented as a colored asterisk for each individual measured. Moreover, the same number of basal bodies within each individual cell were randomly

redistributed and remeasured 1000 times to generate a mean (gray circle) and standard deviation (gray bar) projected at the same height as the corresponding individual (see methods). A Clark-Evans value of 1 indicates a random distribution of basal bodies whereas a Clark-Evans values of 0 and 2 indicate a non-random clumped or gridded distribution, respectively. Thus each individual is compared to a randomized representative of itself and p-values were obtained by a two-sided T-test for all cells within the strain.

Fig S4: Nearest neighbor mean distance (NNMD) histograms and symmetry values for each strain in figure 4. Strains expressing FliM-GFP as a feducial for flagellar basal bodies were imaged by OMX 3D-SIM. Basal body coordinates were determined using Imaris (see methods). (**A**) The nearest neighbor was determined using MATLAB and measured in µm. Data is represented as histograms plotted by binning data for each strain into 20 bins from 0 to 1.5 µm. **(B)** The basal body symmetry over the long axis of the cell was determined as the ratio of higher to lower basal body count relative to the cell midpoint. Data is represented as histograms plotted by binning data for each strain into 20 bins from 0 to 1.5. The following strains were used to generate the data: WT (DK1906), *fliE* (DK5081), *flgB* (DK5082), *flgC* (DK5268), *flhO* (DK5083), *flhP* (DK5084) and IPTG-inducible strain (DK31).

Movie S1: Rare basal body mobility is observed in wild type cells. Timelapse TIRF microscopy of wild type (DK1906) cells expressing FliM-GFP Images were taken every 250ms for 1 minute and played back at 60 frames per second. Arrow indicates mobile basal body.

Movie S2-4: FliM-halo and FliF-mNeongreen colocalize in mobile basal bodies. Timelapse TIRF microscopy of cells expressing FliM-halo (magenta) and FliF-mNeongreen (green) while mutated for the flagellar rod (DB1413). Movie S2 shows the merged timelapse of both channels. Movie S3 shows the green channel only. Movie S4 shows the magenta channel only. White arrow indicates a moving basal body. Images were taken every 250ms for 1 minute and played back at 60 frames per second.

Movie S5: Increased basal body mobility is oberved during early stages of *fla/che* induction*.* Timelapse TIRF microscopy of FliM-GFP in A strain in which the P_{flache} promoter was replaced by the IPTG-inducible $P_{hyspank}$ promoter (DK31). Timelapse was taken after 15 minutes of IPTG induction. White arrows indicate mobile basal bodies. Images were taken every 250ms for 1 minute and played back at 60 frames per second.

Movie S6: Infrequent basal body mobility is observed in cells mutated for *flhG.* Timelapse TIRF microscopy of FliM-GFP in cells mutated for *flhG* (DK2117). Images were taken every 250ms for 1 minute and played back at 60 frames per second. White arrow indicates moving basal body.

Figure S1

A

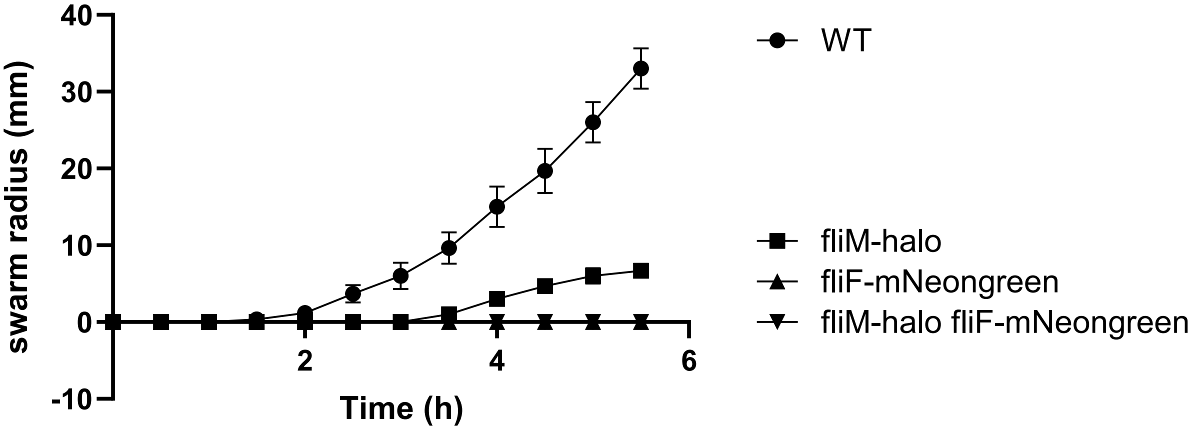

40
30
20
10
0
-10
swarm radius (mm)
2
4
6
Time (h)
WT
fliM-halo
fliF-mNeongreen
fliM-halo fliF-mNeongreen

B

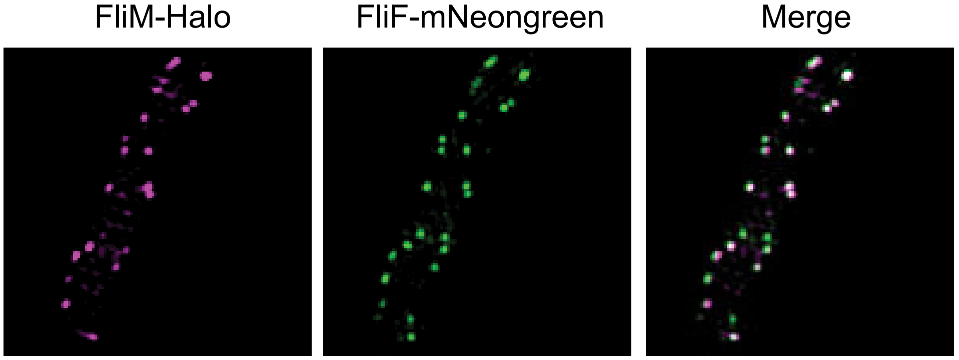

FliM-Halo
FliF-mNeongreen
Merge

Figure S2

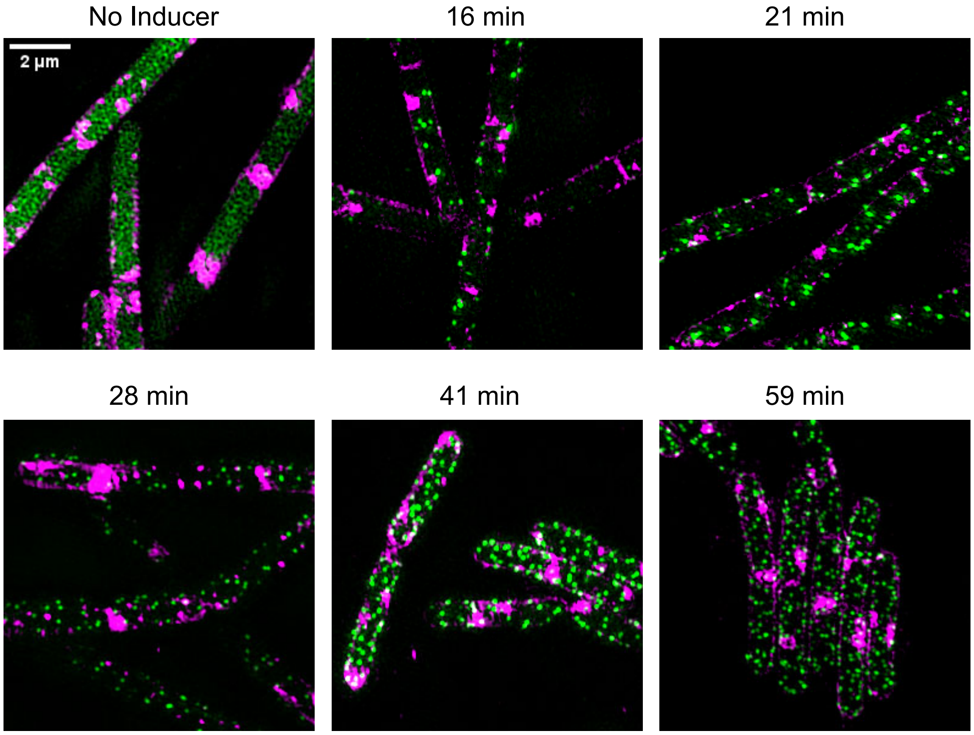

Figure S3

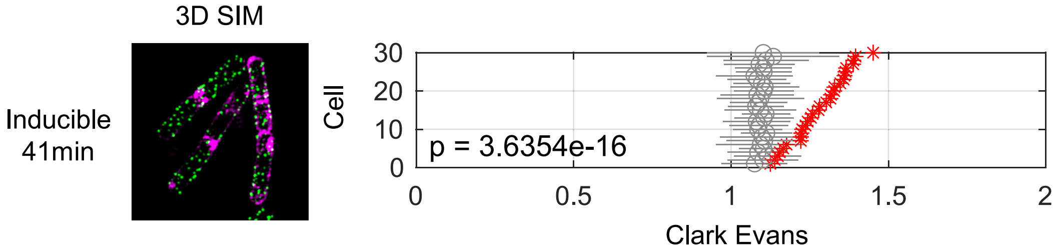

3D SIM
Inducible
41min
Cell
30
20
10
0
p = 3.6354e-16
0
0.5
1
1.5
2
Clark Evans

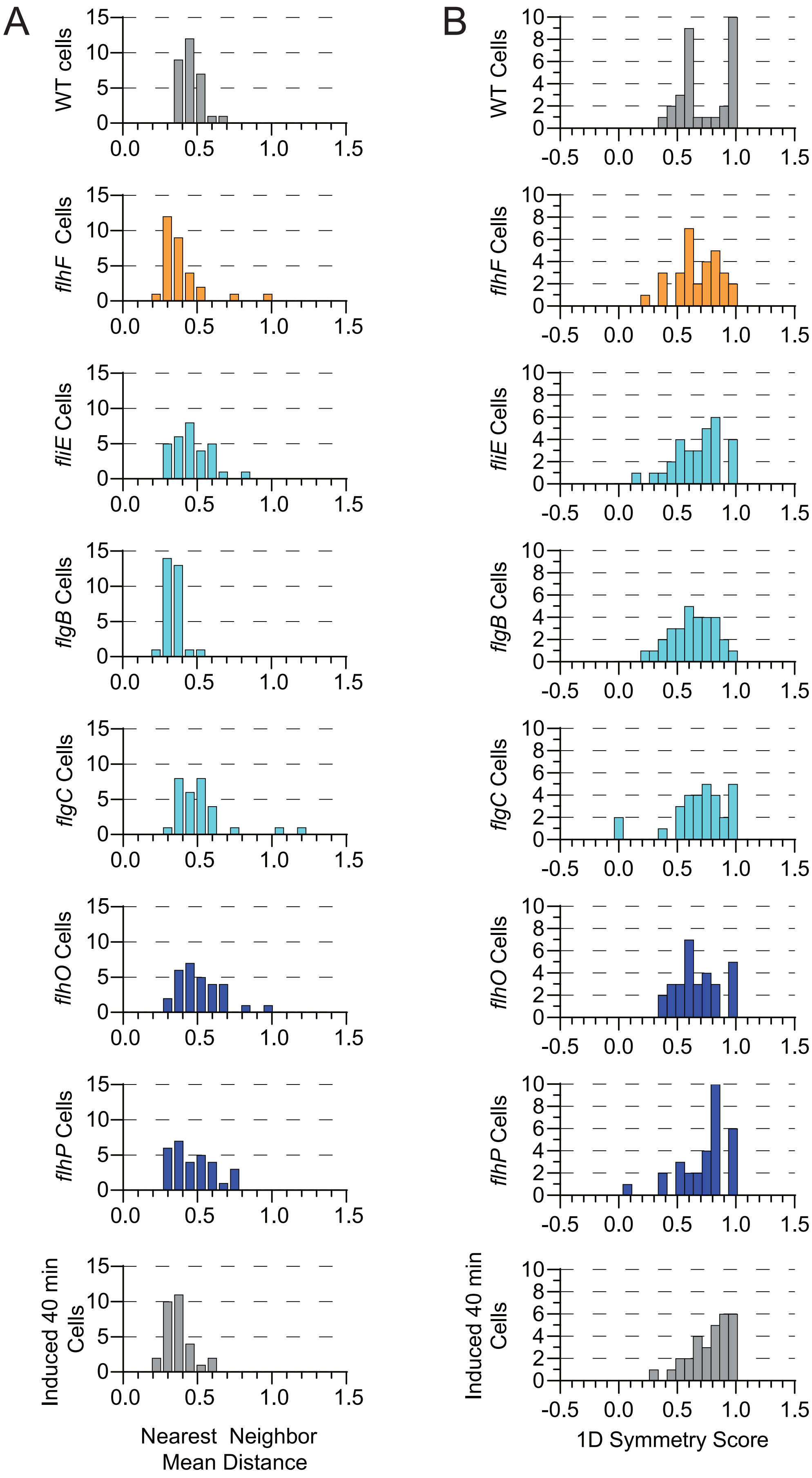
A
B
WT cells
flhF Cells
fliE Cells
flgB Cells
flgC Cells
flhO Cells
flhP Cells
Induced 40 min Cells
Nearest Neighbor Mean Distance
WT Cells
1D Symmetry Score
